# Editorial: Beyond Bariatric Surgery: Expected and Unexpected Long-Term Evolution

**DOI:** 10.3389/fendo.2021.838892

**Published:** 2022-02-16

**Authors:** Yves Schutz, Zoltan Pataky

**Affiliations:** ^1^ Department of Medicine, University of Fribourg, Fribourg, Switzerland; ^2^ Division of Endocrinology, Diabetes, Nutrition, and Therapeutic Patient Education, University Hospitals of Geneva and University of Geneva, Geneva, Switzerland

**Keywords:** obesity, bariatric (weight loss) surgery, psychological outcomes, cardiometabolic outcomes, complications

## Introduction

The increasing demand for the surgical treatment of obesity is in line with the increasing prevalence of obesity worldwide, particularly class III obesity. Nobody will challenge today the well-known beneficial effect of bariatric surgery in obesity treatment, notably the main effect on weight loss and weight-related co-morbidities.

Incidentally, these positive effects are not due to the surgery *per se* but to the weight loss achieved (1), including type 2 diabetic patients who present with obesity (2). There are, in parallel, well-known risk of complications as well as risk related to the voluntary malabsorption procedure which have been described decades ago. The latter progressively leads to a deficit in both macronutrients (protein in borderline supply) as well micronutrients (vitamins, minerals, and trace elements). The degree of deficit of nutrients and the degree of potential nutrient imbalance can vary enormously among patients. The inter-individual variability appears to range from 20 to 100% (3). The nature of surgery intervention as well as endogenous individual factors undoubtedly plays a role.

**Figure 1:**
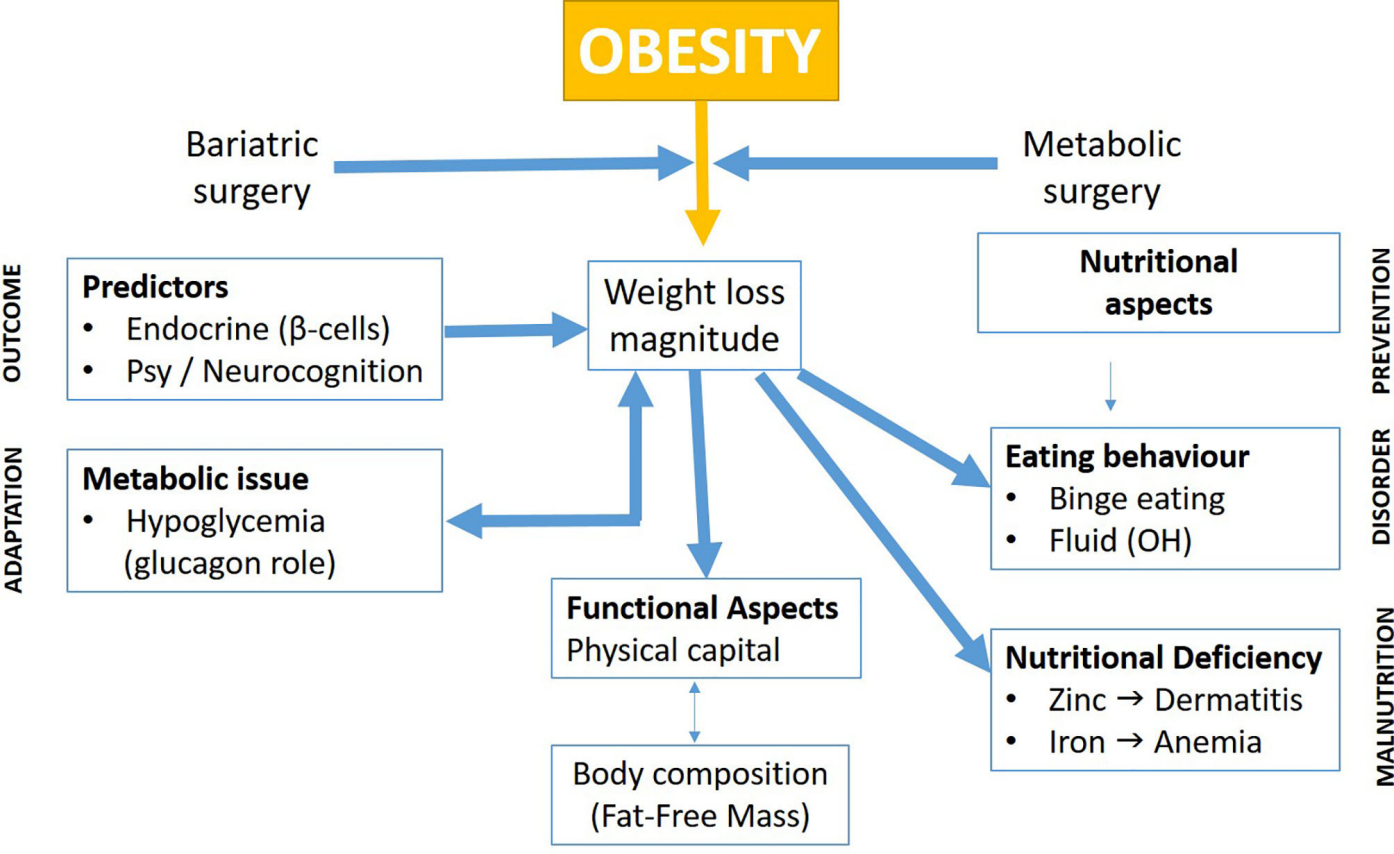
Multidimensional effects of bariatric surgery.

The topics of the present Frontiers in Endocrinology issue gather various contributions highlighting the actual research activities in bariatric surgery. On the first sight, this may appear rather heterogeneous. In order to demonstrate the complementarity of these various bariatric surgery topics, their articulation is depicted in a synthetic global scheme (**Figure 1**), which attempts to show the interrelated field of the research domain presented.

As shown in **Figure 1**, the different topics can be tentatively classified into several headlines which shall be discussed below.

## Predictors of Weight Loss After Bariatric Surgery

These refer to predictors such as (a) cellular/endocrine beta cell functions and (b) psychological/neurocognitive factors (Bianciardi et al.). In their paper, Borges-Canha et al. have shown, in a retrospective longitudinal study of 1,561 non-diabetic patients, that B-cell function, as evaluated by HOMA-beta index at baseline, could be associated to long-term weight loss after bariatric surgery. They showed that patients with worst insulin secretory function tend to lose more weight (Borges-Canha et al.).

Psychological parameters and wellbeing status before surgery are the key factors influencing the outcomes post-surgery. Up to one-third of patients undergoing weight loss surgery are non-responders presenting insufficient weight loss and excess weight regain at mid and long terms. Eating disorders, anxiety, depression, excess of stress, and its inappropriate management are frequent triggers of emotional eating. On one side, these disorders do not represent surgical contraindications, and on the other side, their role in long-term changes in both dietary habits and physical activity is well known and is experienced by the majority of patients.

In their article, Bianciardi et al. highlighted the importance of analyzing the interactions among different factors involved in weight loss outcomes, such demographics, psychopathology, eating disorders, and cognitive functioning. The issue is not to contraindicate the surgery but to better assess and treat patients who present with such conditions (Bianciardi et al.).

## Metabolic/Endocrine Issues

When compared to that of insulin, the role of glucagon, a kind of “forgotten hormone”, to prevent hypoglycemia and the particular importance of the “insulin to glucagon balance” are highlighted (Lobato et al.).

It appears that some complications of bariatric surgery, such as the dumping syndrome and hypoglycemia, may have been often underestimated, although these have been known for decades, when non-obesity-related gastrectomy was performed. Inappropriate secretion of glucagon or its potential mis-adaptation consecutive to bariatric surgery, leading to a too-high insulin to glucagon balance ratio, could explain this phenomenon.

## Eating Behavioral Issues

In line with the above-mentioned predictors of post-surgery weight outcomes, a tailored psychoeducational intervention for patients with a history of binge eating disorders is presented in this issue (Eik-Nes et al.). The better assessment and delivery of this intervention may contribute to better patient selections and to the optimization of psychological follow-up in order to prevent weight regain on the long term. Such patient may need a lifelong psychological follow-up.

The development of alcohol abuse or other addictive behaviors in bariatric surgery patients post-operatively is a possible side effect of the surgery on the mid or long term. A prospective evidence from a US cohort study has shown that adults with severe obesity and who have been undergoing gastric bypass were associated with an increased risk of incident alcohol use disorder symptoms, illicit drug use, and substance use disorder treatment (4). In the present issue, colleagues from Norway demonstrated an increased risk of presumed problematic drinking behavior in women having had alcohol problems prior to surgery. However, there is a need for appropriate screening tools at both pre- and post-surgery (Strømmen et al.).

## Nutritional Deficiencies

Two nutritional factors of key clinical importance to monitor after bariatric surgery have been presented, namely, zinc (Herrera-Martínez et al.) and iron (Sandvik et al.). In the first study, the authors discuss a case study of the clinical sign of dermatitis in a woman at 1 year after biliopancreatic diversion surgery, which was fully explained by moderate malnutrition and, specifically, zinc deficiency. In the second study, the authors pointed out that many patients progressively developed iron deficiency anemia after a decade or more following Roux-en-Y gastric bypass surgery. This corroborates the importance of long-term iron nutritional status surveillance before surgery and for a lifetime follow-up in the post-surgery period.

## Changes in Integrative Body Functions

Consecutive to bariatric surgery, physical capacity and body composition change in terms of fat-free mass *vs*. fat mass losses and the relationship between these 2 factors. In their article, our Swiss colleagues have pointed out the importance of an exercise program aimed to maintain fat-free mass and to enhance muscle strength and balance post-surgery (Reinmann et al.). There is a need to develop a consensus on such physical rehabilitation and reconditioning programs tailored for bariatric surgery patients in order to prevent excessive fat-free mass loss related to both rapid and massive weight loss.

We hope that the readers will appreciate the diversity of papers in this Research Topic covering bariatric surgery as useful references for state-of-the-art bariatric surgery research. This merits additional human research which would take into account the variability in response of operated patients (both inter-individual and intra-individual) at any stage in the post-surgery follow-up period.

## Author Contributions

All authors listed have made a substantial, direct, and intellectual contribution to the work and approved it for publication.

## Conflict of Interest

The authors declare that the research was conducted in the absence of any commercial or financial relationships that could be construed as a potential conflict of interest.

## Publisher’s Note

All claims expressed in this article are solely those of the authors and do not necessarily represent those of their affiliated organizations, or those of the publisher, the editors and the reviewers. Any product that may be evaluated in this article, or claim that may be made by its manufacturer, is not guaranteed or endorsed by the publisher.
